# Medical Students’ Perceptions of a Blockchain-Based Decentralized Work History and Credentials Portfolio: Qualitative Feasibility Study

**DOI:** 10.2196/33113

**Published:** 2021-10-22

**Authors:** Anton Hasselgren, Katina Kralevska, Danilo Gligoroski, Arild Faxvaag

**Affiliations:** 1 Department of Neuromedicine and Movement Science Norwegian University of Science and Technology Trondheim Norway; 2 Department of Information Security and Communication Technology Norwegian University of Science and Technology Trondheim Norway

**Keywords:** blockchain, eHealth, qualitative research, VerifyMed

## Abstract

**Background:**

Increased digitization of health care might challenge some of the trust functions that are established in a traditional health care system. We have, with the concept of VerifyMed, developed a decentralized service for work history and competence verification, as a means to increase trust in the virtual interaction between a patient and a caregiver, mitigate administrative burden, and provide patient-reported outcomes seamlessly for health professionals.

**Objective:**

This research aimed to validate the use case of a decentralized credentials service for health care professionals in Norway. We also aimed to evaluate the proof-of-concept of VerifyMed, a blockchain-based credential service for health care professionals.

**Methods:**

A qualitative approach was applied with data collection through 9 semistructured interviews and 2 focus groups (one with 4 participants and the other with 5 participants). The System Usability Scale (SUS) was used as a part of the interviews. Data were analyzed through the principles of systematic text condensation. The recruitment of participants ended when it was concluded that the data had reached saturation.

**Results:**

The following 5 themes were identified from the interviews and focus groups: (1) the need for aggregated storage of work- and study-related verification, (2) trust in a virtual health care environment, (3) the potential use of patient feedback, (4) trust in blockchain technology, and (5) improvements of the VerifyMed concept. The SUS questionnaire gave a score of 69.7.

**Conclusions:**

This study has validated the need for a decentralized system where health care professionals can control their credentials and, potentially, their reputation. Future work should update the VerifyMed system according to this input. We concluded that a decentralized system for the storage of work-related verifiable credentials could increase trust in a virtualized health care system.

## Introduction

### Background

The COVID-19 pandemic has accelerated the digital transformation of the health care sector. Social distancing and other measures to reduce transmission of SARS-CoV-2 have forced health systems to deliver health services using innovative methods [1]. Virtual health care consultations, which often are referred to as telemedicine, are an example of this transformation that has had a rapid increase during the pandemic. Telemedicine visits increased by 683% in New York City during the spring of 2020 [2], and general practitioners in Norway reported that 81% of them used video consultation during the pandemic (most of them did not use it at all before the pandemic) [3]. Since the advantages of telemedicine include cost-effectiveness, increased access, and availability [4], we can assume that this increase will be permanent. In previous work, it was suggested that telemedicine might challenge some of the established structures for trust in a patient–health care professional relationship [5]. The ability to verify the competence of health care professionals will be of increasing importance in telemedicine in order to enhance trust [6,7].

The administrative burden placed on health care professionals has perhaps always been present [8]. However, the administrative burden related to work mobility seems to have increased recently [9], and this trend is also reflected in increased mobility among health care professionals [10]. As a result, the administrative burden of verifying credentials and experiences among this working group is increasing.

For the last decades, there has been a focus on putting patients in the center of evaluating clinical care, combined with biomarkers of health improvements [11]. As a mean for this, patient-reported outcome measures (PROMs) have been introduced to measure patient-reported outcomes (PROs). PROs are referred to as the patient’s health, quality of life, or functional status associated with health care or treatment [12]. PROMs are the tools to measure PROs, which could, for example, be a measure of the quality of life. To complement PROMs, patient-reported experience measures (PREMs) have been introduced as a tool to measure patients’ experiences with health care or health services, often with a satisfactory score [12]. PROs may have increasing importance as a means of learning and improving health care professionals, as well as a way for health care professionals to verify their work history [13].

We have identified a need for a new decentralized service for work history and competence verification as a means to increase trust in the virtual interaction between a patient and a caregiver, mitigate administrative burden, and provide PROs seamlessly for health professionals. This concept is described in the next subsection.

### VerifyMed

**Figure 1:**
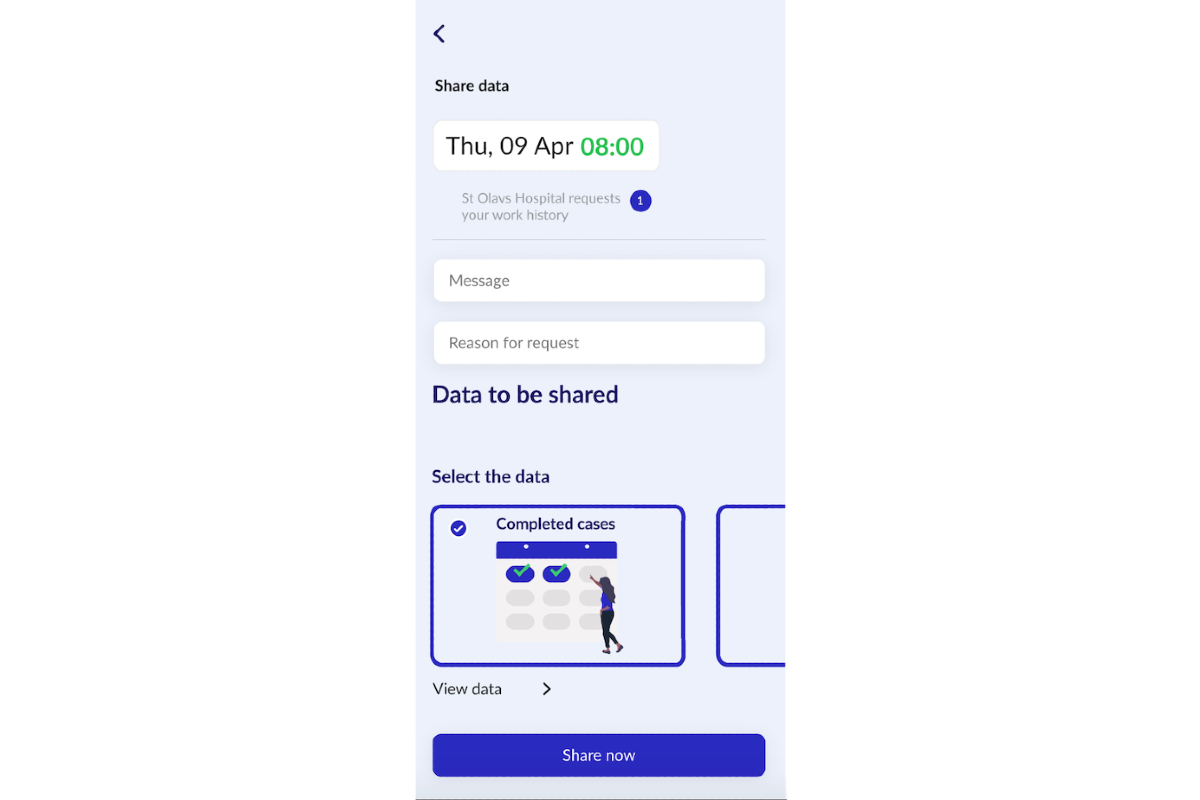
The data sharing page of the VerifyMed user interface.

**Figure 2:**
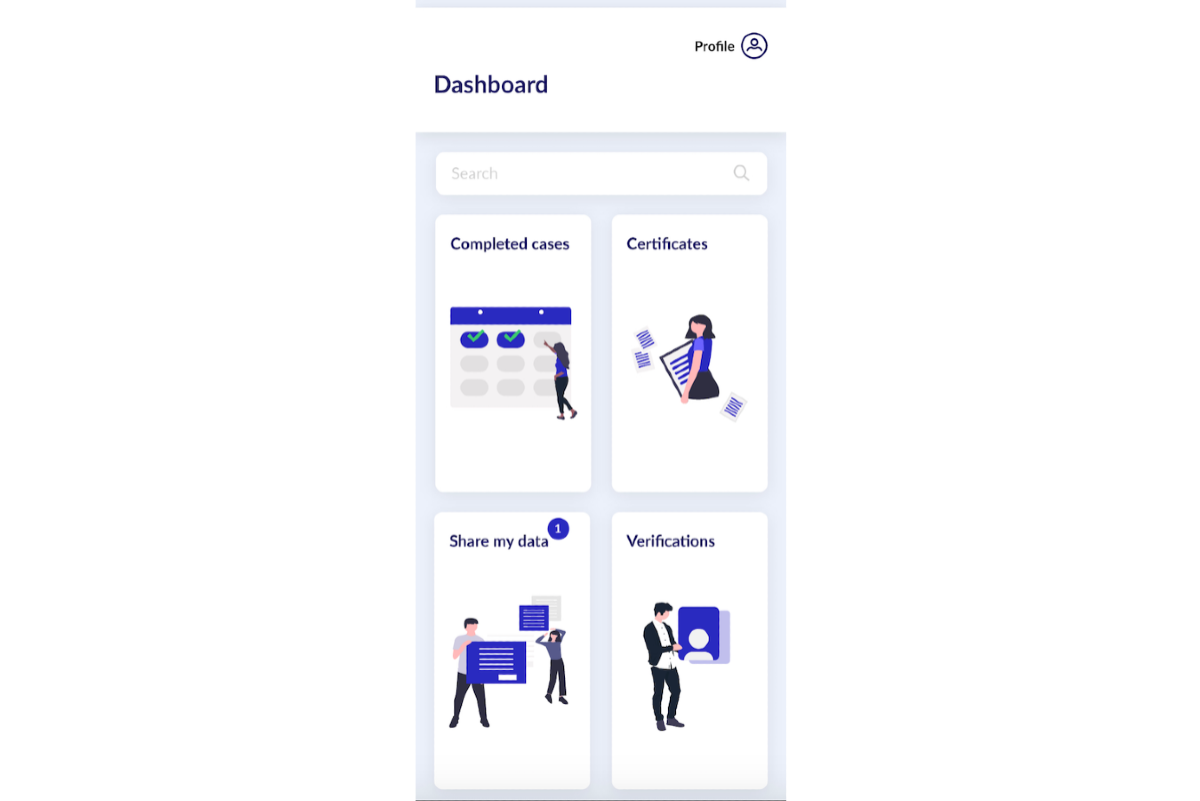
The dashboard page of the VerifyMed user interface.

The proposed concept of VerifyMed provides a solution for enhancing trust between a caregiver and a patient within a virtualized health care environment. The cornerstone of this architecture is an approach for capturing the trust relationships within the health care system by utilizing a blockchain. This trust mechanism can be used by patients to confirm the credentials and potentially enhance their trust in a caregiver during their interaction. Furthermore, the architecture includes a mechanism for evaluating these interactions publicly on the blockchain, using PROMs and PREMs. These evaluations serve as a portfolio of the caregiver’s experience and could potentially be used as a mechanism for continued learning among health care professionals. The concept of VerifyMed is presented further in other reports [5,14,15]. To achieve the objectives of this research, a mock-up of the user interface of the platform was designed using user-centric design theory [16]. The mock-up can be accessed online [17]. Figure 1 and Figure 2 illustrate examples of the user interface that was explored in this research.

### Significance

It is important to gain user input early in technology development to further improve an application according to the needs of the users [18]. After identifying a problem in the health sector and designing a solution for that problem with a proof-of-concept, a feasibility study further validates the concept of VerifyMed and provides valuable user input for further development.

### Aim and Objective

The objective of this work was 2-fold. First, the work aimed to validate a use case of a decentralized medical professional credentials service by mapping out the need for such a service in Norway. Second, we aimed to evaluate the proof-of-concept of VerifyMed, a blockchain-based credential service for health care professionals. We limited the scope of the work to medical students, as they might experience challenges with recording and managing their credentials and experience as they start and progress through their careers.

### Research Questions

The research questions were as follows:

1. What are the potential scenarios of usage from the user group?
2. How will a decentralized system, such as VerifyMed, be accepted by future health care professionals?
3. Does the VerifyMed system meet the requirements of health care professionals who would be using the system?
4. What are the desired features of the users?
5. What are the opinions on a patient-feedback system?

## Methods

### Overview

To answer the research questions, a qualitative approach was applied. Data were collected through 9 semistructured interviews by using the System Usability Scale (SUS) [19] as a starting point for the interviews. In addition, 2 focus groups (one with 4 participants, and the other with 5 participants) were also conducted. The recruitment of participants ended when it was concluded that the data had reached saturation [20].

### Data Collection

Medical students in Norway in study years 4 to 6 were recruited through student organizations. Two focus groups were performed prior to the individual interviews. The focus groups functioned as workshops where blockchain technology [21] and the concept of VerifyMed were presented by the moderator (AH) before any discussion. The moderator asked the participants to describe the current procedures they had experienced with skill verification, certificates, and trust in a virtual health care scenario. Finally, an open discussion on how the presented technology could be used to improve the current workflows was initiated. The focus groups were limited to 45 min. In addition to the moderator, a research assistant was present to take notes.

The duration of the individual interviews was limited to 30 min. The participants were invited to the mock-up of the VerifyMed user interface by an online link. They accessed the mock-up through their web browsers on their laptops. After a short introduction, each participant was invited to perform several simple tasks in the prototype and was asked to explain his or her thoughts during this exploration phase, with minimal assistance from the moderator. The participant was then asked questions from the SUS questionnaire. The SUS is considered to be an easy, quick, and reliable test of usability that is technology agnostic [19]. Based on the answers, follow-up questions followed in a semistructured form. The focus groups and the individual interviews were conducted in an online format, using Zoom (Zoom Video Communications).

### Data Management

The focus group sessions and the user-testing interviews were audio and video recorded. The recordings were analyzed after the sessions by the main researcher (AH). The 3 other researchers in the project (AF, KK, and DG) also reviewed the recordings when there were doubts by the first researcher. The recordings were stored locally on the first researcher’s computer during the project. The recordings were transcribed and erased after transcription, and the notes from the focus group sessions were compared with the transcripts and then erased.

### Data Analysis

Transcription of the collected data was performed according to the 6 steps of transcription proposed by Azevedo et al [22]. Since the data collection was conducted in Norwegian, an English translation of the used quotations was performed by the main researcher (AH). Data were analyzed according to the principles of systematic text condensation [23]. This procedure consists of the following 4 steps: (1) getting a total impression by reading all the text materials and identifying preliminary themes; (2) identifying meaningful units from both the technical aspects of the VerifyMed service and its use by medical students; (3) abstracting condensates from each group and subgroup; and (4) creating synthesized descriptions of the user’s experiences and opinions about the use of a decentralized work history portfolio. The software NVivo (version 1.4.1; QSR International) was used for the analysis.

### Ethical Considerations

All participants were asked to give written consent based on oral and written information about the study. Only those who gave their consent to participate in the study, according to the information in the consent form, were included (n=9). The study did not collect or otherwise handle patient- or health-related data. Therefore, ethical clearance from the Regional Ethical Committee (REK) was not obtained. The study was registered by NSD - Norwegian Center for Research Data and the Data Protection Officer at the Faculty of Medicine and Health Science (Norwegian University of Science and Technology) to be General Data Protection Regulation compliant.

## Results

### Participant Characteristics

A total of 9 participants were recruited in the study, and all 9 completed both participation in the focus group and the individual interview. The characteristics of the respondents are presented in Table 1.

**Table 1:** Characteristics of the informants.

Characteristic		Value (n=9), n (%)
**Gender**		
	Male	4 (44)
	Female	5 (56)
**Age (years)**		
	23-24	3 (33)
	25-26	2 (22)
	27-28	3 (33)
	>28	1 (11)
**Study year (out of 6)**		
	4	1 (11)
	5	5 (56)
	6	3 (33)
**University**		
	Norwegian University of Science and Technology	8 (89)
	University of Oslo	1 (11)
**Previous knowledge of blockchain**		
	No	6 (67)
	Yes	3 (33)

### Themes

The results from the SUS were mainly used as a starting point for the individual interviews. The quantitative results from the SUS were calculated using the standard formula for SUS [24]. The score was 69.7, with fairly equal responses from the respondents. A score above 70 is considered acceptable, according to validation studies [24]. In the data analysis, 5 themes were identified within the focus groups and individual interviews, and an overview is presented in Table 2. The results from both methods of data collection were intertwined. Several of the themes were discussed in both the focus groups and individual interviews, and they are therefore presented here jointly.

**Table 2:** Results overview.

Theme	Proportion^a^ of data	Supporting quotes
The need for an aggregated storage of work- and study- related verifications	24.2%	*...large parts of the system is trust based. I don’t know how to verify certificates, but as you say, paper-based certificates are an easy way to falsify knowledge and experience.*
Trust in a virtual health care environment	26.0%	*To showcase what you have done related to courses and such could contribute, it becomes the equivalent to have diplomas on the wall. It is not necessarily certain that the patient understands what it is, but it can improve the total impression.*
The potential use of patient feedback	14.5%	*The ones who write feedback are the patients how are either very pleased or they who are very displeased. ...the selection gets skewed.*
Trust in blockchain technology	7.3%	*I think I understand the value with that things could be verified and that falsification might be mitigated with time-stamping and such, that I see as positive...*
Improvements of the VerifyMed concept	6.5%	*I envision that in the future, when things get more digital and patients have a specific problem and want to get in contact with a doctor who has done research in that area or has any specific courses within the area...*

^a^The percentages do not add to 100 since other themes, not relevant to the research questions, also were discussed.

#### The Need for an Aggregated Storage of Work- and Study-Related Verifications

The first theme evolves around the need for a platform where medical students can collect and store verifications of their experiences. As the participants describe, as of now, there is no common digital system in use where they can store grades, certificates, references, and verifications of practical assignments. One participant expressed this as follows:

...it would be nice if it could be done digitally. Previously in the studies, we rotated to different departments of the hospital and were supposed to get one signature from each department. We were supposed to keep this piece of paper with over 20 signatures throughout the semester and try to not lose it. It would be an advantage if this could be done digitally.

If this physical paper is lost by the students, they need to collect all the signatures again. This was expressed as a rather common problem and a lot of work. On this occasion, it seems like the supervisors were not always aware of what they approved by giving their signatures.

...if you needed to go back for the signature, it could happen that they were a bit uncertain but most of the time they signed anyway, or always.

The risk of falsified documents with an analog trust-based system was further acknowledged in the discussion. As one respondent expressed:

...large parts of the system is trust-based. I don't know how to verify certificates, but as you say, paper-based certificates are an easy way to falsify knowledge and experience.

#### Trust in a Virtual Health Care Environment

The second identified theme evolves around trust in the interaction between the medical doctor (student) and the patient. Since the respondents were in different stages of their education, they had experienced different exposures to patients. Their perceptions of trust in their encounters with patients also varied. Some respondents did experience a lack of trust towards them among patients.They expressed that this probably was a consequence of they being students and thus being considered less experienced and knowledgeable. However, most of the respondents experienced that trust could be established, and it was not considered a major disadvantage that they were students. Furthermore, trust in a virtual health care environment, mainly video consultations, was discussed. The respondents agreed that this way of providing health services will be an important part of their professional careers. They had so far been exposed to this medium in various degrees, mainly due to COVID-19, where restrictions enforced virtual meetings instead of physical meetings. Their perceptions of quality in virtual health services, compared to physical services, varied. Some experienced no difficulties in gaining the trust and confidence of patients. However, most seemed to agree that the lack of physical attributes and the lack of physical examinations may harm the trust-building mechanisms.

You get something for free in a hospital setting, you walk-in in a white coat, that looks professional. I believe most doctors perform virtual consultation from a setting that looks professional, otherwise, it can look suspicious.

The individual interviews further explored the need for digital verifications, and the general opinions among the respondents were that this could have a purpose in a virtual environment.

To showcase what you have done related to courses and such could contribute, it becomes the equivalent to have diplomas on the wall. It is not necessarily certain that the patient understands what it is, but it can improve the total impression.

However, participants were also hesitant about how this information would be interpreted by the patients, and if they would comprehend the meaning of such certificates and other proofs of competence.

...I’m a bit uncertain regarding this. What value would it bring if they could see this, it might be difficult for them to interpret. It’s difficult to say what they would use this information for.

#### The Potential Use of Patient Feedback

The third theme identified was the expectations and fears around a patient-feedback system, such as PROs. In this discussion, the Norwegian website Legelisten [25], a site where anyone can rate their general practitioner, was referred to several times. The respondents’ general opinions around this service were negative, and the patient-feedback system was associated with the negative impressions of this service. For example, the risk of a biased selection of users of this service was expressed as follows:

The ones who write feedback are the patients who are either very pleased or they who are very displeased. ...the selection gets skewed.

The participants also expressed a general fear of being publicly rated, similar to the rating system of Legelisten [25]:

...agree that it could be an individual asset but nothing that should be published publicly, how good you are in comparison with others because that will create competition rather than provide you with learning.

This fear also extended to how data could be reported in a feedback system. Several respondents expressed the need for this kind of feature to be objective and systematic. Allowing patients to provide feedback without any systematic framework was expressed as being associated with a major risk of information overload and useless information from the patients.

...maybe you should not be able to write free text with no limit and maybe you should limit how the feedback is given, otherwise it will be a lot of irrelevant and unserious feedback, so it has to be a limitation for the patients’ possibility to provide feedback.

A feedback system as a means for health care professionals to learn was however expressed as something positive among the participants. At present, they have little or no opportunity to know the outcome of a given patient treatment, since they often rotate and may miss a revisit or the results when the patient gets referred to another department.

You often wonder how it went and what happened to the patient.

It would be great to get a small correction and feedback on what you have done and how it went, and what conclusions were made further. That would be gold worth to know...

#### Trust in Blockchain Technology

The fourth theme, trust in blockchain technology, was briefly discussed. As Table 1 indicates, a few of the respondents had knowledge about blockchain technology prior to participating in this research. Even though blockchain was introduced in a presentation by the moderator (AH) before the focus group discussions, several of the respondents reported that they did not understand the technology. However, none of them showed any negativity toward the technology and whether to trust the VerifyMed service.

I think I understand the value with that things could be verified and that falsification might be mitigated with time-stamping and such, that I see as positive. But I don’t know enough about the technology to say if it gives any large advantages compared to other services. I think I understand it, but I’m not a technical person.

As expressed by several respondents, the trust in the service was dependent on third-party validation and trust in the developers behind the service. One respondent commented as follows:

Yes, if the source is trustworthy and it helps if it is promoted by persons you trust. ...but if it is an unknown actor which I could not relate to I would be much more skeptical to provide any personal information.

#### Improvements of the VerifyMed Concept

The last main theme that was discussed were general improvements and opinions regarding the VerifyMed user design and features experienced by all respondents. None of the respondents had any problems completing the 9-item task list given, and they all did so in a short amount of time (3-7 min). The general expression was that the solution could be useful and that they acknowledge the need for this kind of service. One respondent commented as follows:

I envision that in the future, when things get more digital and patients have a specific problem and want to get in contact with a doctor who has done research in that area or has any specific courses within the area, then it could be very useful for both the doctor to be able to show knowledge and interests in that particular area, then you might get more patients you can include in your research or that you find interesting.

The informants expressed that the design and user flow were something that they were familiar and comfortable with. They had a few suggestions on improvements and additions of features, such as (1) make it clearer what data are being shared, for how long, and with whom, (2) make it possible to have direct communication with patients through a message system, and (3) make it possible to showcase scientific publications or research projects as a part of the “portfolio.”

## Discussion

### Principal Findings

This research aimed to validate a use case of a decentralized medical professional credentials service by mapping out the need for such a service in Norway and to evaluate the proof-of-concept of VerifyMed, a blockchain-based credentials service for health care professionals.

The informants expressed that the main area of use is a platform where they could store all the data they would need for a job application. This is perhaps an expected result since the respondents are already (or will soon be) in a job-seeking process. The general opinion was that they had no or little control over data, such as verifications of internships or practical assignments, at present. They were all positive about the idea of a system that could automate this and provide them with more control. Presently, it seems to be somewhat up to chance if they receive these paper-based verifications and how useful they are owing to a lack of systematization. This highlights the need for new services with features similar to those of VerifyMed.

Fear was generally expressed for a patient-feedback system among the participants, in case the data are used to evaluate them externally. This fear might be explained by the fact that young physicians (students) are already exposed to a lot of stress and have a fear of making mistakes [26]. The addition of another evaluation service could increase this stress. However, they were generally positive about receiving feedback for their own learning. They were also open to extend this and share the feedback with colleagues and take part in each other’s feedback, for the objective of learning. Previous research has indicated that it might be difficult for health care professionals to learn from patient feedback [27]. The sample in this study (students) might explain this difference, as students are probably more inclined to learn and improve compared to more senior health care professionals. They did however see little or no use in sharing patient feedback with other patients, as they did not see the need for this. This is in line with previous research [28]. The existence of physician-rating websites, such as Legelisten [25], indicates that patients are interested in the feedback of other patients to evaluate physicians. This difference in perception between physicians and patients might again be explained by physicians’ fears of being evaluated and potentially not having control over their reputation as health care professionals. Previous research has indicated that a physician’s reputation on physician-rating websites is critical to attract patients [29], and there seems to be a lack of tools where physicians can take control over their online reputation [30]. This previous knowledge and our results clearly indicate the need for a service where physicians can control their online reputation. Considering this, future updates on the VerifyMed concept should include options to share or not to share patient feedback publicly. This control feature might enhance the acceptability of the service among health care professionals and enable reputation control in a virtualized health care environment.

The quantitative results from the SUS should be interpreted with the understanding that the small sample size prevents any strong conclusions from this quantitative result. However, it could serve as an indicator that the usability of the user design is acceptable [24] (the study showed a SUS score of 70). There were no indications that design changes need to be implemented in the platform based on the user testing.

The limited clinical experience of the informants may have influenced the results, and it is possible that another sample, with more experienced health care professionals, will have other opinions. However, the results from the current informant sample fulfill the objectives of this research. The individual interviews might have been influenced by the discussions in the focus groups and the presentation made by the main researcher (AH), which were both conducted before the individual interviews. The perception of the technology might have been influenced as a result.

### Conclusion

This study validated the need for the concept of VerifyMed, and feedback from the users provided inputs that will further enhance the quality and fit-for-purpose aspect of the concept. Future work should update the system according to these inputs, enhance the data control of the user to provide reputation control, and move to the next step of system development. Furthermore, we concluded that a decentralized system for the storage of work-related verifiable credentials could increase trust in the health system, especially if there are less trusted institutions as a result of an increase in the number of health care providers in a digitally transformed health care system.

## Abbreviations

PREM: patient-reported experience measure
PRO: patient-reported outcome
PROM: patient-reported outcome measure
SUS: System Usability Scale
